# Cabozantinib-associated acquired perforating dermatosis

**DOI:** 10.1016/j.jdcr.2024.06.002

**Published:** 2024-06-14

**Authors:** Denis Smirnov, Hillary Tsibris

**Affiliations:** a Department of Pathology, Brigham and Women’s Hospital and Harvard Medical School, Boston, Massachusetts; b Department of Dermatology, Brigham and Women’s Hospital and Harvard Medical School, Boston, Massachusetts; c Center for Melanoma Oncology, Dana-Farber Cancer Institute, Boston, Massachusetts

**Keywords:** acquired perforating dermatosis, cabozantinib, elastosis perforans serpiginosa, multikinase inhibitor, perforating disorder, tyrosine kinase inhibitor

## Introduction

Cabozantinib is a small molecule tyrosine kinase inhibitor (TKI) that prevents cancer progression and angiogenesis by targeting vascular endothelial growth factor (VEGF) receptor, as well as MET and AXL receptor tyrosine kinases. It is approved for use in several solid tumors, including metastatic renal cell carcinoma (RCC).^1^ Although VEGF pathway blockade selectively and effectively slows tumor growth by inhibiting tumor angiogenesis and largely sparing mature vascular tissues, it also disrupts essential signaling pathways in the epidermis and hair follicles, results in characteristic cutaneous adverse effects.^2^ Hand-foot skin reaction is the most commonly reported cutaneous adverse effect associated with VEGF receptor TKIs.

Less commonly reported cutaneous side effects of other TKIs are acquired perforating dermatoses (APDs), characterized by the transepidermal elimination of dermal material. APDs are commonly are divided into 4 groups, namely elastosis perforans serpiginosa (EPS), reactive perforating collagenosis, perforating folliculitis, and Kyrle disease, although there is some evidence that APDs may occur on a spectrum rather than representing distinct clinical entities.^3^^,^^4^ TKIs may induce APDs through inhibition of pathways involved in normal follicular and epidermal development and differentiation, including platelet-derived growth factor receptor and epidermal growth factor receptor.^5^, ^6^, ^7^ Although APDs have not been reported in association with cabozantinib therapy, APDs with features of both perforating folliculitis and EPS have occurred during treatment with sorafenib, a multikinase inhibitor that, similarly to cabozantinib, inhibits VEGF receptor.^8^ Here, we report a case of an APD with features of EPS that occurred during cabozantinib therapy.

## Case report

A 61-year-old gentleman with a history of metastatic papillary RCC presented to dermatology for evaluation of a 2-year history of recurrent lesions on his bilateral thighs and shins. He had no history of diabetes or renal disease. He had been diagnosed with RCC with pulmonary and lymph node metastases 2 years prior, after presenting with abdominal pain and a bloody cough, at which time a lymph node core needle biopsy confirmed a diagnosis of metastatic papillary RCC. He was initially enrolled in a trial of combination therapy with ipilimumab, nivolumab, and cabozantinib. However, he experienced grade 3 liver toxicity after 1 cycle, and ipilimumab and nivolumab were stopped in favor of continuing cabozantinib monotherapy.

**Fig 1:**
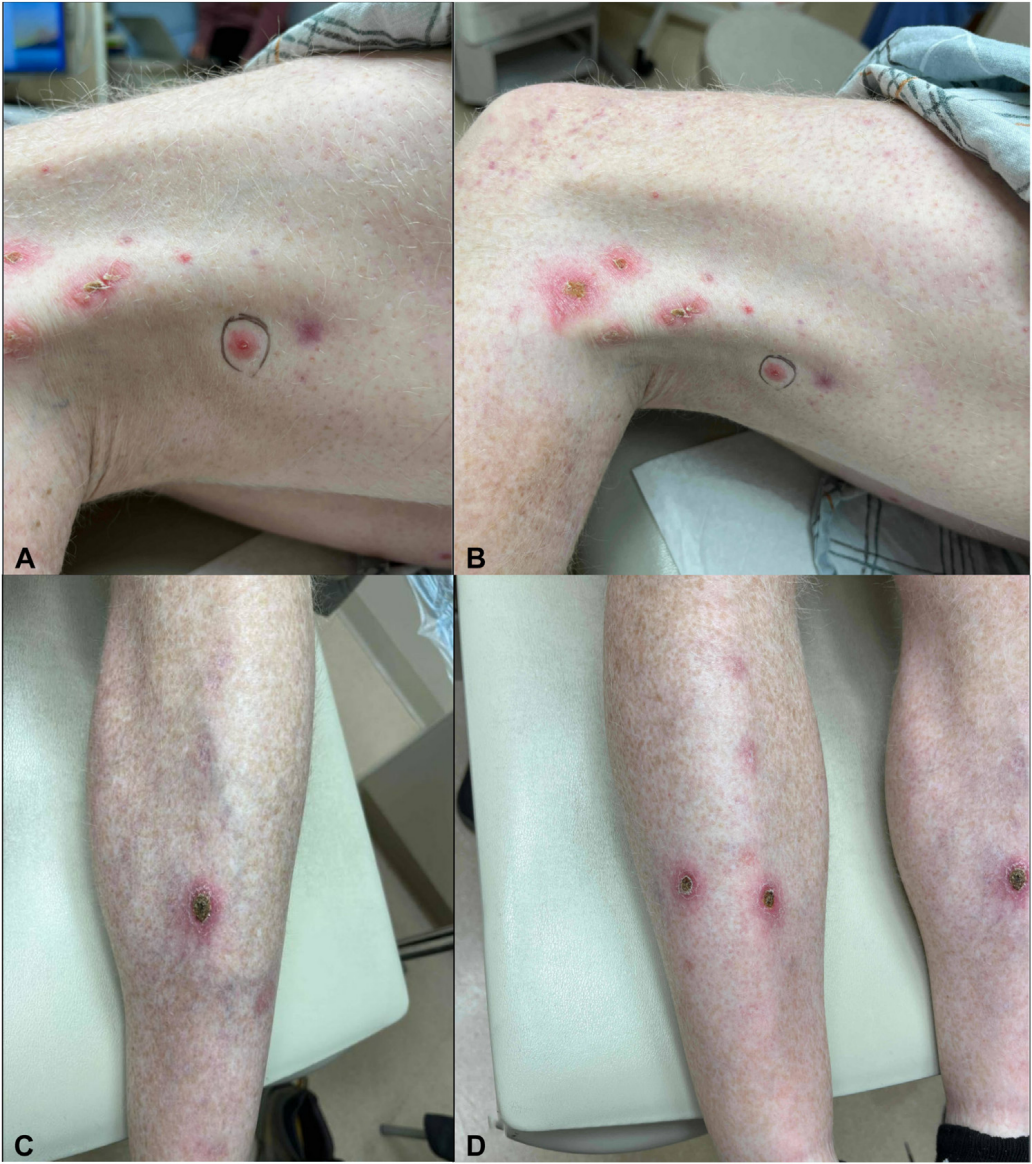
Clinical images showed pink nodules with adherent crust and central ulceration on (**A, B**) the medial thigh and (**C, D**) bilateral pretibial regions. Areas of hypopigmentation at sites of healed lesions are also visible.

**Fig 2:**
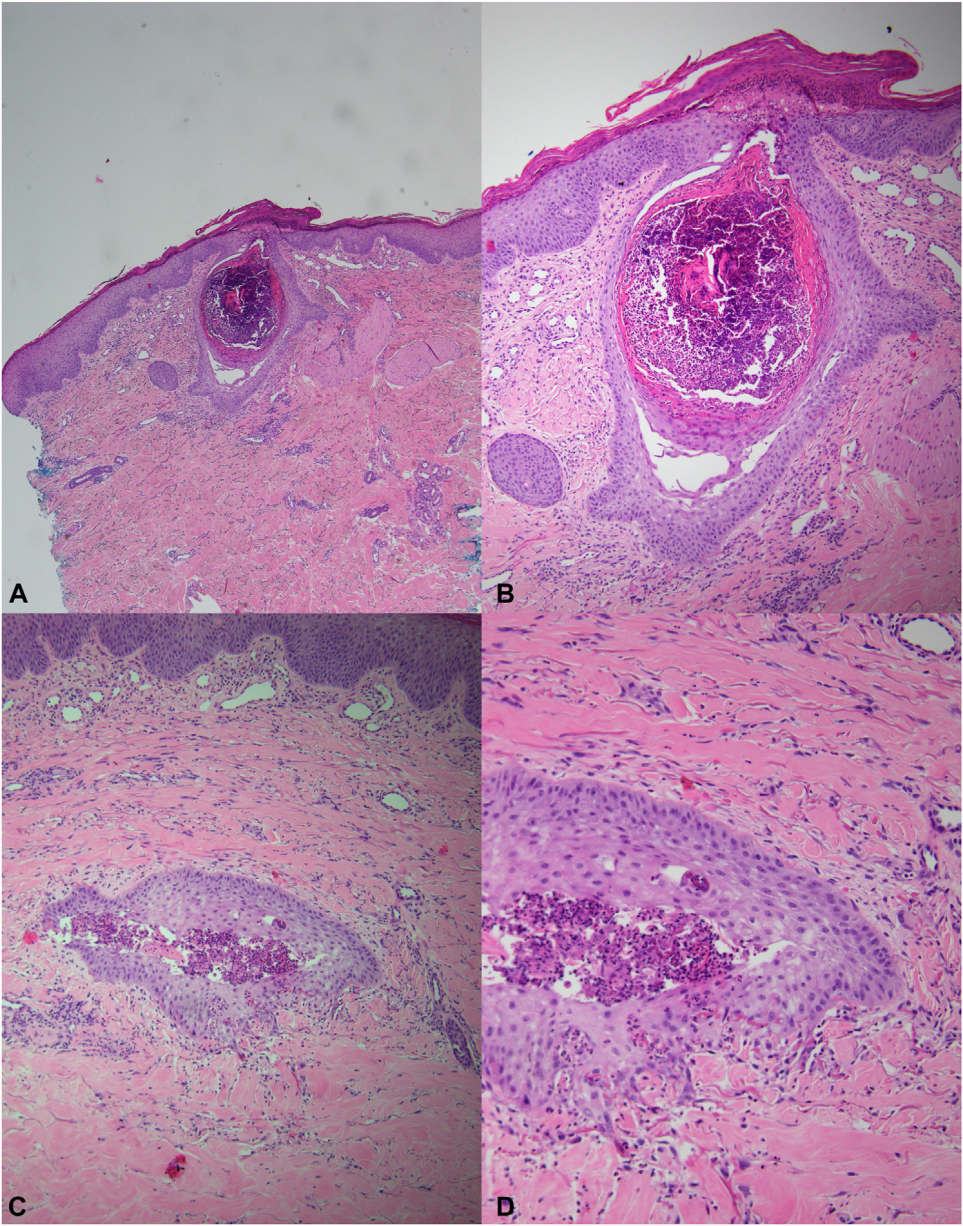
Punch biopsy from the medial thigh showed transepidermal elimination of material on (**A**) low and (**B**) high power H&E. Deeper H&E sections showed elastic fibers within the epithelium of the invagination on (**C**) low and (**D**) high power. *H&E*, Hematoxylin and eosin.

**Fig 3:**
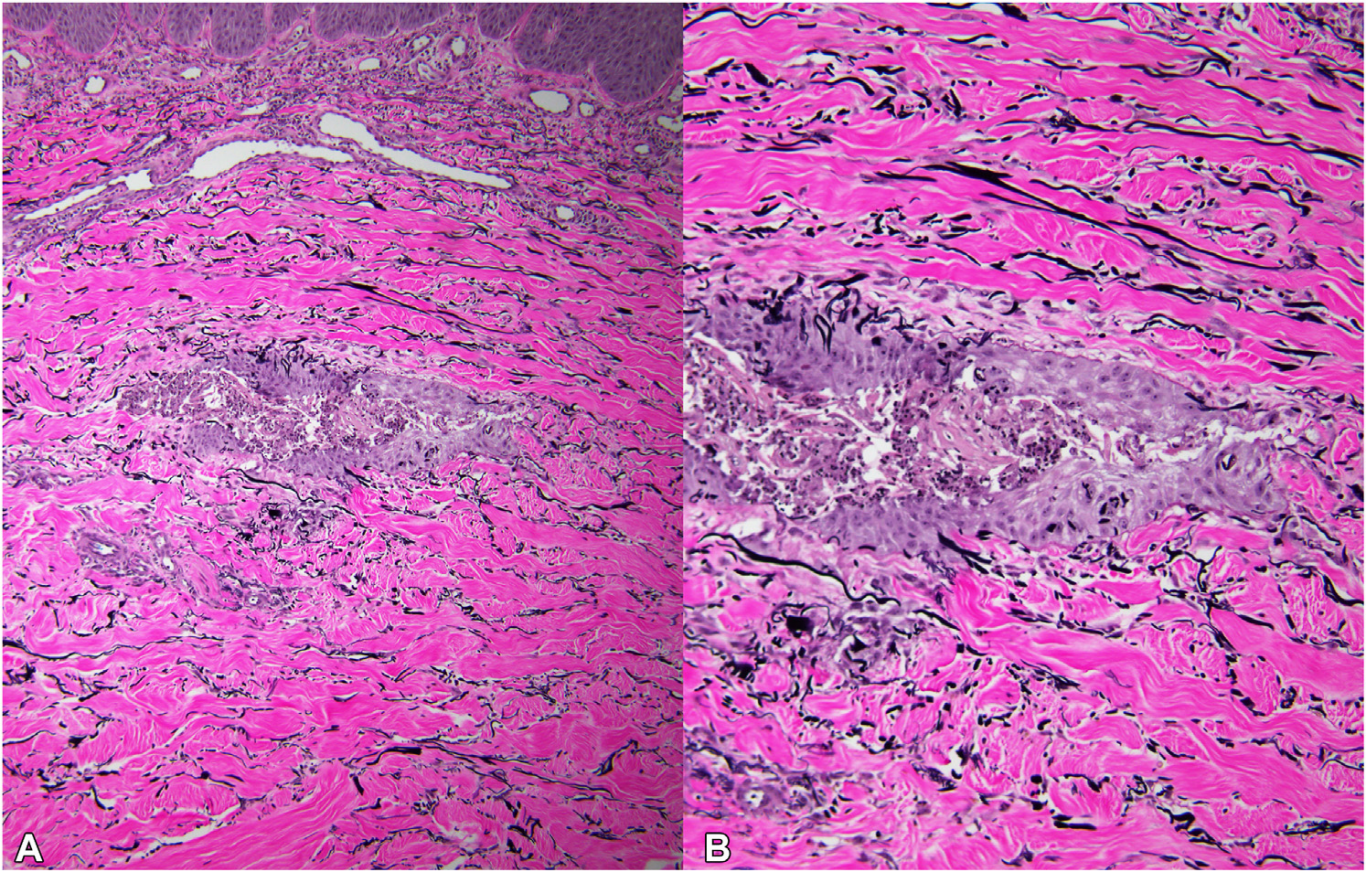
An elastic stain confirmed the presence of elastic fibers within the wall and cavity on (**A**) low and (**B**) high power.

His metastatic disease remained largely stable on cabozantinib, however, he experienced painful and occasionally pruritic skin lesions on his bilateral shins and medial thighs. They appeared spontaneously without preceding trauma and would self-resolve but recur in the same locations. He treated the areas with at-home red light therapy and clobetasol ointment without success and was referred to dermatology for further evaluation. On cutaneous examination, he had pink nodules with adherent crust and central ulceration on the bilateral medial thighs and pretibial regions with hypopigmented macules at sites of previously healed lesions (Fig 1). A punch biopsy was performed for diagnostic confirmation, which showed epithelial invagination with associated reactive and acute inflammatory changes associated with foci of intraepithelial elimination of elastic fibers. This was thought to be consistent with a diagnosis of an acquired perforating disorder with features suggestive of EPS (Fig 2). Elastic stains confirmed the presence of elastic fibers within the epithelial wall and cavity (Fig 3). Gram and fungal stains were negative for microorganisms. The presence of intraepithelial elimination of elastic fibers differentiates EPS from other APD subtypes that exhibit transepidermal elimination of keratin or collagen.

Treatment for an APD was initiated with topical tretinoin. Future treatment options including narrow-band UV-B phototherapy and oral acitretin were also discussed with the goal of limiting cancer treatment interruption. Unfortunately, cabozantinib was discontinued due to disease progression 4 months after APD diagnosis with subsequent improvement in his cutaneous symptoms.

## Discussion

Although APDs have been reported with other TKIs, this case suggests that perforating disorders may occur with cabozantinib therapy as well. The onset of the perforating disorder following cabozantinib initiation suggests that it was triggered by the medication rather than simply the underlying malignancy. The mechanisms by which cabozantinib and other TKIs targeting VEGF receptor may trigger APDs remain unclear.^8^ Inhibition of the VEGF pathway itself may alter endothelial and keratinocyte function and cutaneous wound healing responses, particularly in areas of cutaneous friction or trauma.^9^ TKI-induced alteration in levels of matrix metalloproteinases, which normally degrade elastic fibers, may disrupt elastic fiber homeostasis and result in the transepidermal elimination of elastic fibers seen in EPS.^8^

Greater awareness of the association of TKIs and VEGF inhibition with APDs will hopefully improve clinical recognition and appropriate treatment of these cases, allowing for therapy continuation. Topical steroids and topical retinoids are often used as first-line treatments. Resistant cases may benefit from narrow-band UV-B phototherapy or oral retinoids.^4^ Future studies may help to elucidate whether underlying tumor type, treatment regimen, and preexisting patient factors and comorbidities influence the risk of APD development and clinical subtype.

## Conflicts of interest

None disclosed.
